# Cross-Modality Alignment Perception and Multi-Head Self-Attention Mechanism for Vision-Language-Action of Humanoid Robot

**DOI:** 10.3390/s26010165

**Published:** 2025-12-26

**Authors:** Bin Ren, Diwei Shi

**Affiliations:** 1 Shanghai Key Laboratory of Intelligent Manufacturing and Robotics, School of Mechatronic Engineering and Automation, Shanghai University, Shanghai 200444, China; sdw@shu.edu.cn; 2 Zhejiang Key Laboratory of Robotics and Intelligent Manufacturing Equipment Technology, Ningbo Institute of Materials Technology & Engineering, Chinese Academy of Sciences, Ningbo 315201, China

**Keywords:** Vision-Language-Action (VLA), cross-modality alignment perception, memory-gated filtering attention, multi-head self-attention mechanism, embodied intelligent, humanoid robot

## Abstract

**Highlights:**

**What are the main findings?**

- A model of memory-gated filtering attention was proposed, which improved multi-head self-attention mechanism.
- A cross-modal alignment perception during training was designed, which combined with a few-shot data collection strategy of key steps.

**What are the implications of the main findings?**

- The proposed multi-head self-attention mechanism could reduce the video memory occupation by 72% and improve the training speed from 1.35 s to 0.129 s per batch.
- The proposed Vision-Language-Action of a humanoid robot significantly improved the task success rate and alleviated the robot arm jitter problem.

**Abstract:**

For a humanoid robot, it is difficult to predict a motion trajectory through end-to-end imitation learning when performing complex operations and multi-step processes, leading to jittering in the robot arm. To alleviate this problem and reduce the computational complexity of the self-attention module in Vision-Language-Action (VLA) operations, we proposed a memory-gated filtering attention model that improved the multi-head self-attention mechanism. Then, we designed a cross-modal alignment perception during training, combined with a few-shot data-collection strategy for key steps. The experimental results showed that the proposed scheme significantly improved the task success rate and alleviated the robot arm jitter problem, while reducing video memory usage by 72% and improving training speed from 1.35 s to 0.129 s per batch. This maintained higher action accuracy and robustness in the humanoid robot.

## 1. Introduction

Confronting complex tasks in industrial scenarios is often challenging for embodied intelligent robots, as they require accuracy, generalization across tasks, careful coordination of contact forces, and closed-loop visual feedback [1]. To perform these tasks, the embodied intelligent robot needs not only to master multiple actions but also to execute the appropriate task based on camera images and language instructions, with high accuracy [2]. The manipulation tasks of embodied intelligent robots often involve multiple challenges, including multimodal perception, long-term temporal dependencies, uncertainty in physical interactions, and adaptability to dynamic environments. These features place higher demands on existing data-driven control strategies [3,4].

For robots processing complex long-step tasks, imitation learning (IL) has become one of the important technical approaches by advancing robot operation to actual deployment, as it can directly learn the perception-action mapping from human demonstrations and reduce the costs of complex symbolic modeling and online exploration [5]. Especially in high-dimensional, contact-rich manipulation scenarios, such as insertion, wearing, assembly, and flexible object manipulation, imitation learning is often more feasible and data efficient than reinforcement learning (RL) [6].

However, imitation learning has many limitations. For instance, previous methods [7,8] have demonstrated strong imitation learning performance in open scenarios; however, they still face significant challenges in long-term, multi-step tasks. In particular, one-step BC is prone to compounding errors in multi-step tasks; small prediction biases are gradually amplified over long sequences, leading to failure in the final operation [9,10,11,12]. To address this, recent studies have adopted structured prediction or trajectory-generation mechanisms; for example, combining visual perception with trajectory generation has been explored in robotic arm tasks such as grasping and assembly [5]. Furthermore, experiments with low-cost, low-precision manipulators in unstructured environments show that although imitation learning can achieve initial control with few demonstrations, its robustness decreases significantly as task complexity and continuous control requirements increase [13,14,15].

In recent years, inspired by the breakthrough results of Large Language Models (LLMs) in natural language understanding and reasoning, researchers have gradually introduced language model architectures into the field of embodied intelligence. Attempts have been made to integrate perception, planning, and control capabilities within a unified model framework to improve robots’ semantic understanding and autonomous decision-making in complex, dynamic environments [16,17,18,19,20]. Related work includes both a hybrid architecture (language-as-policy), where an LLM is used for high-level task decomposition and specialized control modules perform low-level actions, and a more tightly coupled closed-loop sense-reasoning-control pipeline. Multimodal observations, such as vision and touch, are continuously fed back to the language module to support online plan revision and action generation [16,17]. In addition, several reviews and empirical studies have shown that combining LLM or multimodal LLM (VLM/MLLM) with world models, retrieval augmentation mechanisms, or security constraint modules can improve generalization and robustness in long-duration tasks, few-shot or zero-shot scenarios, but real-time performance, security, and physical constraint alignment are still major challenges [18,19,20].

In summary, the contributions of this paper to the abovementioned work were as follows:

(1) For the model algorithm, we improved the multi-head self-attention mechanism [21] and proposed memory-gated filtering attention, which not only reduced the video memory occupation but also significantly accelerated the training speed with improved performance and reduced the algorithm complexity.
(2) In terms of datasets, the current end-to-end collection strategy collected all trajectories of the manipulator from the starting point to the target point [22], and there were many noises and redundancies in the intermediate trajectory set. We designed and collected data on key steps, such as the task start point and goal point of the mechanical claw reaching the manipulated object, which can suppress the mechanical arm’s jitter during model inference and enable anthropomorphic operation.
(3) In the training strategy, in order to deal with the sudden addition of a new environmental observation [11] after the robot performs an action each time, which could cause the robot to move unsteadily, we proposed cross-modal alignment sensing. For each key observation frame, the model not only learns to predict the single action strictly corresponding to it but also aligns all possible subsequent remaining action sequences.

## 2. Vision-Language-Action (VLA) for Improved Multi-Head Self-Attention Mechanism

### 2.1. Memory-Gated Filtering Attention

**Figure 1:**
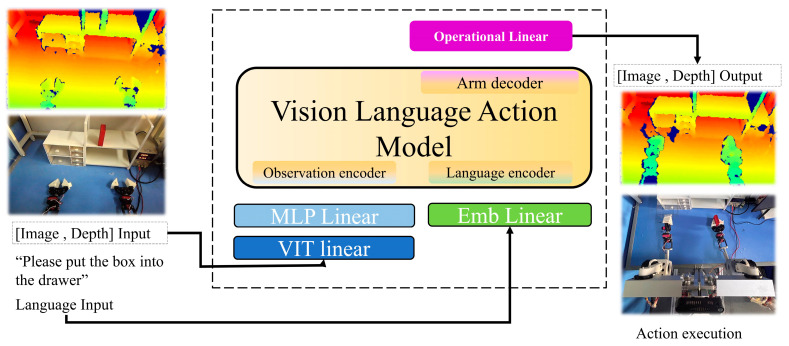
The operation of Vision-Language-Action (VLA).

In the robot operation task, the VLA operation could receive observation information from the environment and a natural-language instruction as input. After multimodal encoding and fusion, the corresponding action sequence was generated for robot execution, the overall process shown in Figure 1.

**Figure 2:**
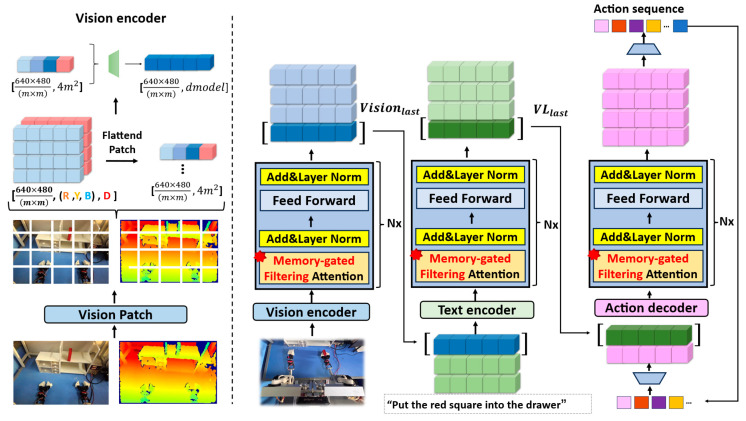
Memory-gated filtering attention model.

To train the memory-gated filtering attention Vision-Language-Action (VLA) model based on a multi-head attention mechanism, we designed a unified encoding process for multi-modal observation data, ensuring that information from the visual, language, and action channels were fully aligned and interacted in the high-dimensional semantic space. The transformer architecture was served as the core processing framework for individual modalities [23], illustrated in Figure 2.

Among them, self-attention was the core mechanism of the transformer model. However, the self-attention algorithm had two particularly obvious shortcomings. The first one was the problem of high computational complexity caused by parallel computing. Its time complexity was $O(N^{2}\times D_{k}$).

In each head, the query matrix $q_{i}$ was first dotted with a learnable parameter matrix $W_{f}$, and the sigmoid function was used for activation to generate the gating filter matrix $sigmod\left(q_{i}*W_{f}\right)$. Subsequently, the row-wise accumulated $k_{i}$ matrix $cosum\left(k_{i}\right)$ undergoes element-wise multiplication (Hadamard product) with this gating matrix for filtering. Finally, the output matrix $sigmod\left(q_{i}*W_{f}\right)⊙cosum\left(k_{i}\right)$ was concatenated with the query matrix $\;q_{i}$, and the combined result was fused through a linear layer $W_{o}$ to obtain the ${\mathrm{Mem}}_{i}\;$ matrix for each head. The outputs of all heads were then concatenated together to yield MemAttn, as shown in the following equation:

(1) $$\;{\mathrm{Mem}}_{i}=[q_{i}\;|\;sigmod\left(q_{i}*W_{f}\right)⊙cosum\left(k_{i}\right)]*W_{o}$$

(2) $$\;\;\;\;\mathrm{MemAttn}=\mathrm{cat}\left[{\mathrm{Mem}}_{1},{\mathrm{Mem}}_{2},\ldots ,{\mathrm{Mem}}_{h}\right]$$

Among them, $W_{f}\in R^{n\times \frac{d}{h}},W_{o}\in R^{\frac{2d}{h}\times \frac{d}{h}}$, for the training parameters, $cosum\left(k_{i}\right)=k_{1}+\ldots +k_{i,\;}\;\mathrm{MemAttn}\in R^{n\times d},q_{i},k_{i}\in R^{n\times \frac{d}{h}}$, n for sequence length d as the feature dimension, *h* is the number of heads.

### 2.2. Multi-Modal Fusion of Vision-Language-Action

First, we could take an observation, each of which consists of an RGB image with a resolution of 640 × 480 and a depth map perfectly aligned with its pixels. In particular, depth information was normalized during preprocessing to ensure stable depth value distributions across different scenes. The natural language instructions corresponding to the task were used as conditional information to participate in subsequent cross-modal interactions. In the input construction stage, the RGB image with size 3 × 640 × 480 and the depth map with size 1 × 640 × 480 were concatenated along the channel dimension to obtain a complete 4 × 640 × 480 RGBD observation tensor.

To further introduce local spatial perception and reduce the input dimension, we adopted the same Patch Embedding strategy as Vision Transformer (ViT) [24]. This was performed as follows:

(1) Each observation data was divided into multiple image patches of fixed size, and the size of each patch was 4 × m × m, containing data of four channels of [R, G, B, Depth].
(2) Each patch was flattened into a one-dimensional vector in the spatial dimension, with a size of 1$\;\times \;4{\;m}^{2}$.
(3) Therefore, a total of $\frac{640\;\times \;480}{\left(m\;\times \;m\right)}$ patches can be obtained for the observed data with a resolution of 640 × 480.

After patch flattening, the representation of the whole frame of observation data becomes:$\;\mathrm{Vision}\_\mathrm{vit}\left[\frac{640\;\times \;480}{\left(m\;\times \;m\right)},4{\;m}^{2}\right]$.

Then, the representation was fed into the Multi-Layer Perceptron (MLP) linear layer, and dimension transformation and feature projection were performed to map each patch into a unified semantic space, yielding the final visual input tensor. Where $d_{model}$ represents the representation dimension defined in the model.

$$\mathrm{Vision}=Linear\left(\mathrm{Vision}\_\mathrm{vit}\right)$$

(3) $$\mathrm{Vision}=\left[v_{1},v_{2},\ldots ,v_{d_{model}}\right],\mathrm{shape}=\left[\frac{640\times 480}{\left(m\times m\right)},d_{model}\right]$$

After the observation data underwent Patch Embedding and MLP projection, the resulting visual representation was fed into a visual encoder module based on a transformer architecture for feature interaction with spatial context. In this stage, the visual sequence was interacted with multi-layer memory-gated filtering attention to preserve the contextual semantic information across local and global perception. Considering the design characteristics of memory-gated filtering attention, only the feature representation of the last time step should be retained as a compact representation of the whole frame observation data in the output of the whole visual encoder, which is denoted as:

(4) $$Vision_{last}\in R^{1\times d_{model}}$$

This representation aggregated the multi-scale information of all patches in the current frame as a high-dimensional semantic embedding of the environment state at that instant.

In the multi-modal fusion stage, the $Vision_{last}$ output from the visual encoder and the corresponding natural language instructions were jointly input into the language encoder. The language encoder also leverages the pre-trained parameters of the large language model to fully exploit the model’s knowledge transfer capabilities for natural language understanding and to enhance its adaptability across diverse task descriptions and scene contexts. Let a given instruction be l, which is passed through tokenizer and embedding to obtain a sequence of word vectors $e=\left[e_{1},e_{2},\ldots ,e_{t}\right]$.

For cross-modal alignment, the visual global feature $Vision_{last}$ was inserted at the top of the sequence and concatenated with the language feature sequence as follows:

(5) $$\left[\;Vision_{last},e_{1},e_{2},\ldots ,e_{t}\right],\mathrm{shape}=\left[1+t,d_{\mathrm{model}}\right]$$

Then, the concatenated multimodal sequences were sent to the language encoder, and through interactions within the multi-layer transformer block, the deep fusion and complementarity of vision-language information are further realized. Finally, only the output of the last time step after fusion was retained as a compact representation of the cross-modal global context semantics, denoted as follows:

(6) $$\;\;\;\;\;\;\;\;\;VL_{last}\in R^{1\times d_{model}}$$

After the previous steps of visual language instruction processing, the next step is action generation. For action, we have seven degrees of freedom for one arm and 14 degrees of freedom for two arms in total, and each action data is denoted as $a_{t}=\left[\;a_{1},a_{2},\ldots ,a_{14}\right]$. In an observation, we have k steps of action. Before processing, we need to project the action using an MLP linear layer to increase the dimensionality.

(7) $$\;\;a_{d}=Linear\left(a_{t}\right)$$

(8) $$a_{di}=\left[\;a_{di1},a_{di2},\ldots ,a_{{\mathrm{di}}_{\mathrm{model}}}\right],a_{d}\in R^{k\times d_{model}}$$

Similarly, we concatenated the output of the last time step of the visual language to the sequence head in the action data to be sent to the action decoder for training, labeled as follows:

(9) $$\;\left[\;VL_{last},a_{d1},a_{d2},\ldots ,a_{dk}\right],shape=\left[1+k,d_{model}\right]$$

The fused visual-language action data $\left[\;VL_{last},a_{d1},a_{d2},\ldots ,a_{dk}\right]\;$ was input to the action-generation decoder. We also used a memory-gated filtering attention module based on a pre-trained large language model for sequence modeling and action generation. By introducing a memory enhancement mechanism, the module establishes stronger context dependence across different action time steps, thereby improving the consistency and stability of multi-step control signal generation in complex operation tasks.

## 3. Cross-Modal Alignment Awareness Strategy

### 3.1. Data Collection for Imitation Learning

In the data collection phase of a robot manipulation task, we can collect a motion trajectory that matches the natural language instruction l, the observed data I within  t∈N during task execution, and the corresponding action sequence $a_{t}$ [25,26]. The existing end-to-end acquisition strategies are generally used to record the complete action trajectory of the robot from the starting state to the completion of the task in a time interval. For example, for the instruction “let the robot open the drawer”, the data collection often covers the whole process from identifying the position of the drawer, approaching, grasping to the drawer being completely pulled open, that is, the collection of the continuous action sequence $a_{t}=\left[a_{0},\ldots ,a_{r}\right]$ [27,28] under the given language instruction l, when the time period $t=\left[t_{0},t_{r}\right]$.

However, in practice, we found that such a long time window trajectory acquisition method often introduces more noise data [29,30], and may lead to the lack of continuity of the generated joint control commands in the model inference stage, which will lead to problems such as joint jitter and trajectory offset during the execution process [31,32]. For the above problem, for example, under the given language instruction l, in the action sequence $a_{t}=\left[a_{0},\ldots ,a_{i},\ldots ,a_{r}\right]$, the key actions are $a_{i}$ and $a_{r}$; then we only need to collect $\;a_{i}$ and $a_{r}$, and the collected action sequence $a_{t}$ becomes $a_{t}=\left[a_{i},a_{r}\right]$. The intermediate action sequences $\left[a_{0},\ldots ,a_{i}\right)$ and $\left(a_{i},\ldots ,a_{r}\right)$, without model inference, only need to let the manipulator reach the key position by itself.

The manipulator will perform multiple action adjustments to the environment before grasping. The mechanical claw’s posture data at the turning point in each path adjustment and the posture information at the final grasp are the “key steps” we need to collect during the task data-collection stage. The figure below shows two simple examples of collecting action-sequence data while the robot grasps a cube, compared to the existing way of working. The following figure shows a simple example.

**Figure 3:**
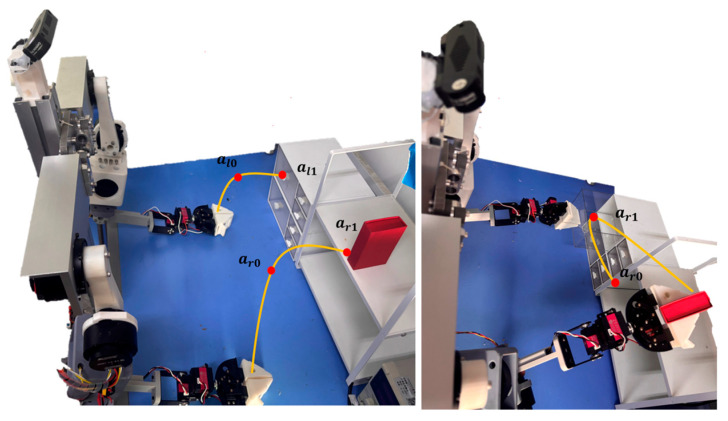
The strategy is collected for key steps after grasping.

After grasping, the robot needs to perform further specific interactive behaviors, such as putting the object into the container, performing insertion operations, or completing dumping. This stage also faces many challenges, especially in tasks with limited operating space or high-precision docking; a single action is often insufficient to complete the entire interaction. Therefore, the robot needs to dynamically adjust the end-effector’s posture and motion path in response to environmental feedback during operation. The image below shows the robot dropping the squares into an open drawer, shown in Figure 3.

The proposed strategy could effectively reduce noise from redundant actions, ensure continuous controllability of joint states, and reduce the complexity of motion planning and generation, thereby improving the robustness and interpretability of robot operations driven by natural language commands.

The observation data I consists of multimodal sensor information, specifically including a frame of RGBD image data of size 640 × 480. Each frame of observation is captured by a single depth camera and contains depth maps that are fully aligned with the color image, ensuring consistent encoding of the scene’s color and geometric information in the same spatial reference frame $I=cat\left[Image,depth\right]$.

**Figure 4:**
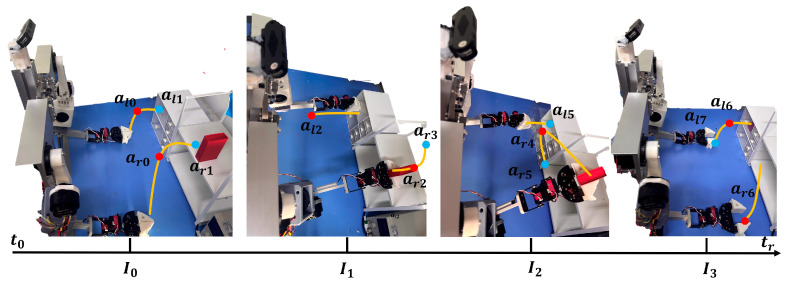
Visual action key steps data collection method.

For sampling observation data, it is necessary to balance these two effects. We adopted the observation update strategy every two key steps; that is, we can use one frame of observation data to guide action generation for two consecutive key steps, thereby significantly improving overall task completion rate while maintaining environmental adaptability and action consistency. It greatly reduces the jump phenomenon of the action sequence at the observation insertion point and makes the robot’s behavior more coherent and natural during the execution of complex tasks, shown in Figure 4.

Through this phased, event-driven observation data-collection method, the robot can plan actions appropriately while maintaining efficient perception of the environment state, thereby better realizing sequential operation and real-time adaptation under complex instructions.

### 3.2. Cross-Modal Alignment of Datasets

After collecting all the natural language instructions l, observations I, and action sequences $a_{t}$ we need to align them for training. Our alignment strategy is to assume that we have collected a set of observation $\;\mathrm{data}\;I_{i}=\left[I_{i0},\ldots ,I_{im}\right]$ and action trajectory data $a_{i}=\left[a_{i0},\ldots ,a_{in}\right]$ under the current natural language instruction $l_{i}$. $I_{i}$ observation data and corresponding relationship with the trajectory $a_{i}$ for $I_{i0}\to \left[a_{i0},a_{i1}\right]$, $I_{i1}\to \left[a_{i2},a_{i3}\right]$,..., $I_{im}\to \left[a_{in-1},a_{in}\right]$, where the correspondence between *n* and *m*, *n* = 2*m* + 1. In order to further improve the consistency of action generation and the coherence of multi-step actions in the inference stage of the model, we no longer uses the data alignment strategy that only aligns the observed data with the corresponding action one by one in the existing work, because the sudden addition of a new environmental observation after each step may lead to the unstable motion of the robot. So, we design the observation-action pairing strategy for cross-stage action alignment.

Specifically, in the training phase, for each key observation frame, the proposed VLA model not only learns to predict the single action strictly corresponding to it but also aligns all possible subsequent remaining action sequences. For example, the first observation corresponds to the entire sequence of actions $\left[a_{i0},a_{i1},\ldots ,a_{in}\right]$, the second observation corresponds to the rest of the sequence except for the first two steps $\left[a_{i2},a_{i3},\ldots ,a_{in}\right]$, and so on. This method effectively enhances the model’s ability to grasp the global task structure during reasoning, helps to generate smoother and continuous control instructions, and avoids mutation or jitter in the action sequence.

To ensure consistent tensor dimensions between the input and output in batch processing, padding must be applied to action sequences with different lengths. However, unlike the padding mask commonly used in NLP tasks, simply ignoring the padding term in the loss function may lead to non-physical action predictions in the continuous robot control scenario. Therefore, in this paper, the “standby action” $a_{p}\;$ after the task is completed is introduced as a semantically reasonable filling value for the action length when alignment is insufficient. Specifically, if the length of the valid action sequence corresponding to an observation frame is less than the maximum length, it will be filled with several $a_{p}$ at the end of the sequence, which is formally expressed as:

(10) $$\;I_{it}\to \left[a_{i2t},a_{i\left(2t+1\right)},\ldots ,a_{in},a_{p},\ldots ,a_{p}\right]$$

where $a_{p}$ represents the stable standby state of the robot after the task is completed, which can physically ensure the execution continuity and avoid unnecessary action jitter or unstable output. In this way, the alignment of the observation data and the motion trajectory is as follows:

(11) $$\left[\begin{array}{c} \begin{array}{c} I_{i0}\\ I_{i1} \end{array}\\ I_{i2}\\ \vdots \\ I_{im} \end{array}\right]=\left[\begin{array}{cccccc} a_{i0} & a_{i1} & a_{i2} & a_{i3} & \ldots & a_{in}\\ a_{i2} & a_{i3} & a_{i4} & a_{i5} & \ldots & a_{p}\\ a_{i4} & a_{i5} & a_{i6} & a_{i7} & \ldots & a_{p}\\ \vdots & \vdots & \vdots & \vdots & \ddots & \vdots \\ a_{in-1} & a_{in} & a_{p} & a_{p} & \ldots & a_{p} \end{array}\right]$$

## 4. Complex Task Design of Humanoid Robot

### 4.1. Complex Task

**Figure 5:**
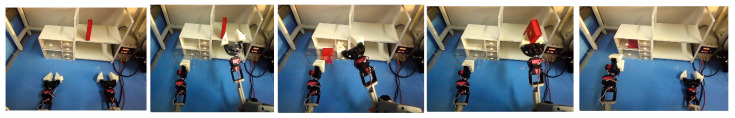
Task 1: put the red square into the drawer [Supplementary Materials: Video S1].

**Figure 6:**
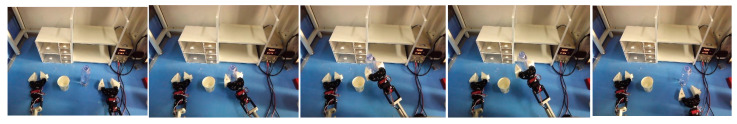
Task 2: pour water.

**Figure 7:**
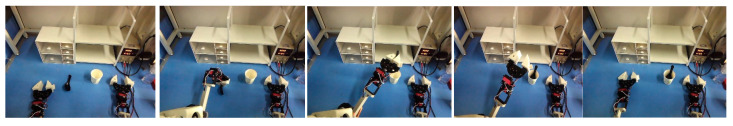
Task 3: spoon into cup.

**Figure 8:**
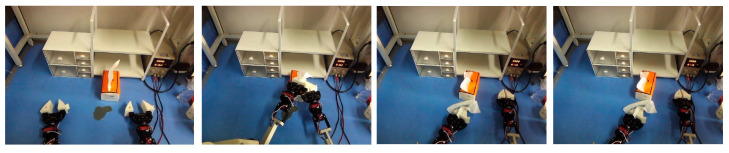
Task 4: surface cleaning.

**Figure 9:**
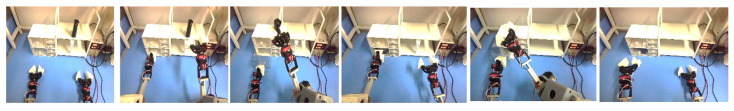
Task 5: classified storage of materials.

**Figure 10:**
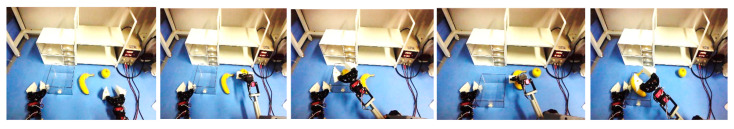
Task 6: sorting fruit.

**Figure 11:**
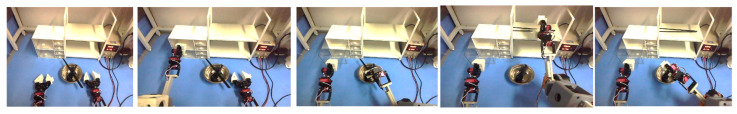

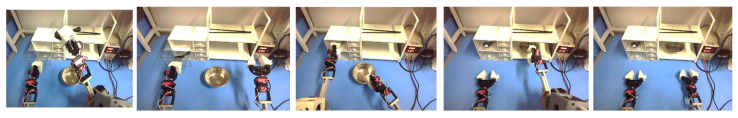
Task 7: arrangement and placement of utensils.

To systematically evaluate the generalization ability and execution accuracy of the proposed VLA model on complex, multi-stage dual-arm manipulation tasks, we designed and selected seven common and challenging robotic tasks that could cover multiple types of object manipulation, spatial coordination, and cross-modal information fusion. Each task required the cooperation of two manipulators and high demands on language understanding, spatial perception, and fine control. These tasks included placing objects, opening and closing drawers, grasping targets, liquid dumping, and container operation, shown in Figure 5, Figure 6, Figure 7, Figure 8, Figure 9, Figure 10 and Figure 11. The descriptions of task segmentations were as follows:

(1) “Put the red square into the drawer.” The shelf has two layers; the red square is randomly placed on the first or second shelf. (subtask #1) The left arm opens the drawer (subtask #2), the right arm locates the red square and grabs it (subtask #3), the right mechanical claw places the square into the drawer (subtask #4), and the left arm closes the drawer.
(2) “Pour the water from the bottle into the cup.” There is a bottle full of water in front of you, and an empty paper cup. (subtask #1) Right arm looks for and grabs the bottle with water in it (subtask #2), picks up the water bottle and approaches the paper cup (subtask #3), evenly pours the water from the cup into the paper cup (subtask #4), and right arm puts the cup back in place after pouring the water.
(3) “Put a spoon into a cup,” with a spoon in front of an empty paper cup. Only one spoon can be placed in the cup (subtask #1). The left arm locates the spoon and grasps it (subtask #2). It stops over the mouth of the cup without any error (subtask #3), puts the spoon into the cup, and raises and draws back the arm (subtask #4).
(4) “Wipe the table with paper towels.” There is a paper towel on the table with a random, irregular stain. When pumping the paper towel, both arms need to stay close together to prevent the paper towel from being lifted. (Subtask #1) The left arm looks for the tissue and holds it, while the right hand presses the paper bag (subtask #2). The left arm removes the tissue and moves to the stained area, and the right hand returns to its original position (subtask #3). Left arm repeatedly wipes the stained area until it is clean.
(5) “Put garbage bags and drugs into different drawers”. There are drugs on the first shelf layer, and garbage bags on the second. (Subtask #1) The left arm locates and grasps the drawer handle on the first floor with the, and the right arm is raised to the position where the garbage bag can be grasped. (Subtask #2) The left arm opens the drawer and keeps it open. The right arm picks up the trash bag and brings it to the top of the first drawer layer. (Subtask #3) After the right arm places the garbage bag in the middle of the drawer, it locates the medicine. (Subtask #4) Robot locates the top drawer on the left, then grabs the medicine with the robot’s right arm. (Subtask #5) It pulls the drawer open with the left arm, lifts the right arm to the top of the drawer, and places it in the middle. (Subtask #6) The left arm closes the drawer, and the right arm returns to the initial position.
(6) “Put the specified fruit into the box”. There are boxes on the left side of the table, and bananas and apples on the right. (Subtask #1) The right arm is instructed to choose whether to grab an apple or a banana. (Subtask #2) The right arm places the specified fruit into the box.
(7) “Store bowls, chopsticks, and spoons in shelves and drawers”. Bowls with chopsticks and spoons inside are placed on the table. (Subtask #1) Robot positions the left arm and grabs the handle of the first drawer. (Subtask #2) Robot opens the drawer with the left arm and keeps it open. It positions the right arm above the chopstick and grabs it. (Subtask #3) It puts the chopsticks on the top shelf with the right arm. (Subtask #4) Then it moves the right arm over the spoon in the bowl. The spoon is easy to move in the bowl, so it needs to be grasped slowly. (Subtask #5) After picking up the spoon with its right arm, the robot moves it to the top of the drawer. (Subtask #6) The right arm needs to accurately place the spoon into the drawer and withdraw the right arm. (Subtask #7) Robot closes the drawer with the left arm, moves the right arm to the position where the bowl can be grasped, and grasps the bowl. (Subtask #8) The left arm closes the drawer, withdraws, and returns to the initial position, and the right arm picks up the bowl and delivers it to the first position on the shelf. (Subtask #9) The right arm releases the mechanical claw to lower the bowl and then withdraws to the initial position.

### 4.2. Comparison of Experimental Results

In this paper, we compared the proposed method with three mainstream baseline methods—RDT [33], OpenVLA [34], and Aloha [35]. Our model was trained in a PyTorch Version 2.1.0 environment with an 8 GB NVIDIA RTX 4060 graphics card. We increased the model parameter size to 500 MB and the pre-trained model size to 122 MB.

Then, we applied the pre-trained model to our model for 10,000 iterations. Pretrained large language model batch size is 512, MemVLA model batch size is 1024, Optimizer: AdamW, pretrained large language model learning rate is 1 × 10^−4^, MemVLA model learning rate is 1 × 10^−3^, dimension size is 1024. The MemVLA model loss function is L1loss.

We adopted the success rate as the main metric, defined as the number of successful trials divided by the total number of trials. Each scenario was tested 25 times. To understand the model’s capability boundaries and error types during actual execution, we decomposed each task into multiple operational sub-stages and analyzed the model’s performance on these key sub-stages. This evaluation method not only focused on the overall task’s success but also emphasized fine-grained decision-making throughout the process. Specifically, each task typically consists of an object recognition phase, grasp phase, operation or transmission phase, fine interaction stage.

Object recognition phase: Locate the task goal, such as finding the correct drawer, item, or target container. Grasp phase: The manipulator is controlled to reach the target position and complete the firm grasp. Operation or transmission phase: The grasped object is moved to the target position, and the path rationality and attitude are controlled. Fine interaction stage: high-precision interactive actions such as inserting, dumping, and closing the drawer, shown in Table 1, Table 2, Table 3, Table 4, Table 5, Table 6 and Table 7.

To verify the advantages of the proposed lightweight self-attention mechanism in terms of resource efficiency, we systematically compared it with the standard fully connected self-attention method under a completely consistent training environment (including hardware platform, optimizer configuration, batch size, and training data). The evaluation metrics include GPU memory usage and the average time per training round.

The proposed method greatly speeds up training while significantly reducing video memory usage, effectively alleviating the computational and memory bottlenecks of the self-attention mechanism in long-sequence scenarios. As shown in Table 8, we compare the memory usage and training delay of the two attention mechanisms under the same settings, which verifies the superiority of the lightweight design.

**Figure 12:**
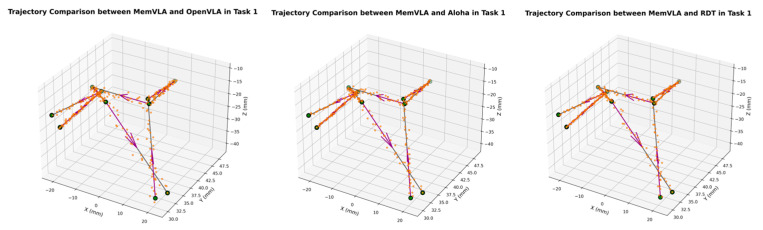
Comparison of trajectories between MemVLA, OpenVLA, Aloha, and RDT in Task 1.

**Figure 13:**
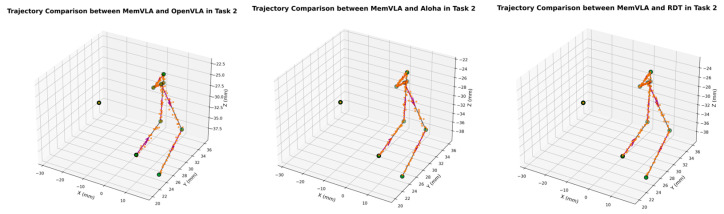
Comparison of trajectories between MemVLA, OpenVLA, Aloha, and RDT in Task 2.

**Figure 14:**
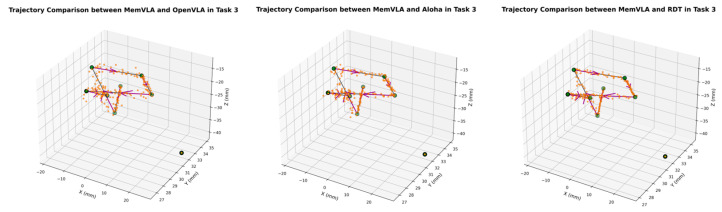
Comparison of trajectories between MemVLA, OpenVLA, Aloha, and RDT in Task 3.

**Figure 15:**
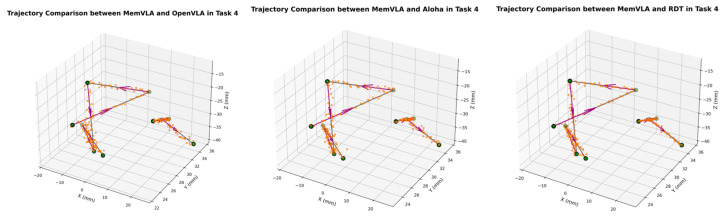
Comparison of trajectories between MemVLA, OpenVLA, Aloha, and RDT in Task 4.

**Figure 16:**
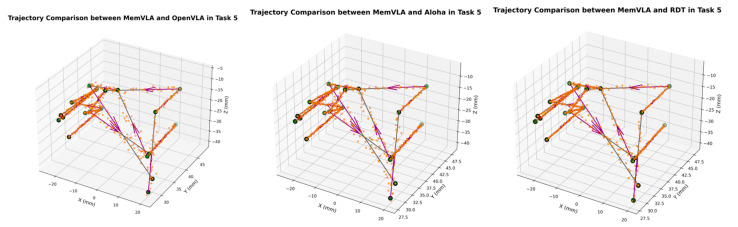
Comparison of trajectories between MemVLA, OpenVLA, Aloha, and RDT in Task 5.

**Figure 17:**
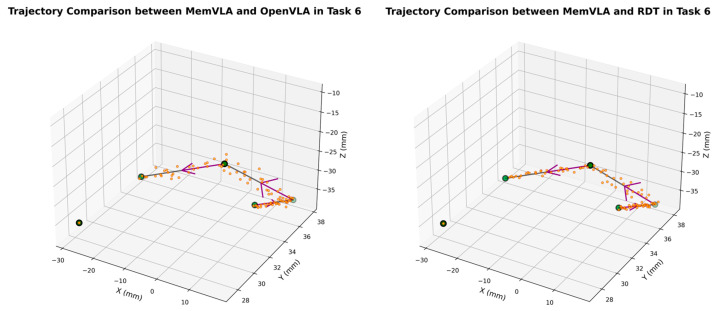
Comparison of trajectories between MemVLA, OpenVLA, Aloha, and RDT in Task 6.

**Figure 18:**
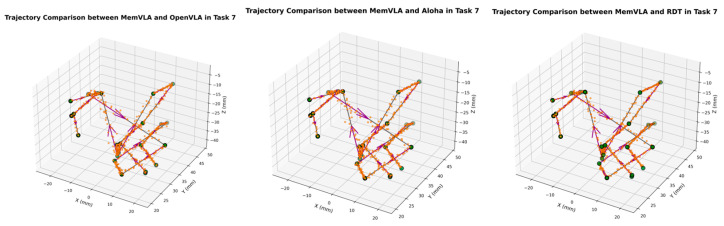
Comparison of trajectories between MemVLA, OpenVLA, Aloha, and RDT in Task 7.

The end-effector trajectory diagrams for the proposed method and other approaches are shown in the following Figure 12, Figure 13, Figure 14, Figure 15, Figure 16, Figure 17 and Figure 18, which compares trajectories from OpenVLA, Aloha, and RDT in sequence. In the figure, the orange endpoints represent the trajectory endpoints output by existing SOTA methods, while the green endpoints denote that output by the proposed method. The solid lines indicate the motion trajectories of the proposed method. It should be noted that, in motor control, we did not perform interpolation; instead, the motors were commanded directly to reach key waypoints, resulting in solid lines. The arrows indicate the direction of the end effector’s gripper movement.

From the above analysis, it can be observed that state-of-the-art (SOTA) methods such as OpenVLA, Aloha, and RDT exhibit significant jitter in robotic arm task execution, with this phenomenon predominantly concentrated between defined key action points. The core of this issue lies in their inherent inference mechanism: analogous to the reasoning logic of large language models, these methods require sequential inference and decision-making for each individual action. Consequently, the generated action sequence $\left[a_{0},\ldots ,a_{i},\ldots ,a_{r}\right]$ struggles to maintain motion continuity. In contrast, the ideal trajectory of a robotic arm should be a smooth straight line or curve; thus, the discretely inferred action points fluctuate around it, leading to considerable trajectory variance.

Delving into the fundamental causes, jitter primarily stems from two core factors: first, the teleoperation bias at the dataset level. If teleoperators fail to skillfully and precisely control the robotic arm via the joystick, the collected action sequence data will inherently contain significant jitter. Subsequent deep learning models, when fitting such flawed data, further amplify this intrinsic defect. Second, the inherent limitations of deep learning itself. Models trained using deep learning can only approximate the collected motion trajectories to within finite error, rather than achieving perfect alignment. This approximation error ultimately manifests as jitter in actual robotic motion.

The MemVLA model introduced targeted improvements to the inference mechanism: the model only needs to infer and generate key action points. During execution, the robotic arm merely requires accurate positioning at these key nodes, while the action sequences between consecutive key points are autonomously executed by the robotic system without additional model inference. This design inherently suppressed robotic arm jitter at the root of the reasoning logic, ensuring smooth trajectories.

## 5. Conclusions

This paper proposed a visual–language action-generation model for fine-grained manipulation tasks. By combining an efficient dual-arm collaboration strategy with a lightweight memory-gated filtering attention mechanism, a multimodal task dataset covering seven typical two-hand fine manipulation tasks was constructed. Through detailed evaluation at the task and sub-stage levels, we demonstrated the model’s stability and versatility in handling multi-stage collaborative tasks. We can apply the trained multi-head self-attention mechanism to the automatic handling of automotive interior parts in practice.

Our training method offered significant advantages in data efficiency, the autoregressive generation strategy, and task completion rate. Specifically, with memory-gated filtering attention, we have reduced the memory footprint by 72% and sped up training by an order of magnitude (from 1.35 s per batch to 0.129 s) while maintaining higher motion accuracy and robustness during critical task phases. The proposed VLA model could provide a more efficient and scalable technical path for large-scale, high-complexity robot task execution in future.

## Figures and Tables

**Table 1:** Comparison of put the red square into the drawer task of utensils completion rates.

	Open Upper Drawer Position	Grab Parts Box	Precisely Place	Drawer Close	Total
OpeVLA (%)	10	0	0	50	0
Aloha (%)	50	80	10	70	10
RDT (%)	68	88	77	89	65
MemVLA (%)	100	90	90	100	90

**Table 2:** Comparison of pour water task of utensils completion rates.

	Locating Liquid Containers	Grab the Container and Move It	Fill Up Liquid	Put the Bottle Back	Total
OpeVLA (%)	50	0	0	0	0
Aloha (%)	88	100	37	73	37
RDT (%)	100	100	77	89	77
MemVLA (%)	100	100	90	100	90

**Table 3:** Comparison of spoon into cup task of utensils completion rates.

	Grab the Spoon	Place the Spoon into the Container	Stable Withdrawal	Total
OpeVLA (%)	74	40	74	40
Aloha (%)	100	80	100	80
RDT (%)	90	100	100	90
MemVLA (%)	100	95	100	95

**Table 4:** Comparison of surface cleaning task of utensils completion rates.

	Identify and Locate the Paper	Pull Out a Paper Towel	Wipe the Table	Total
OpeVLA (%)	80	0	0	0
Aloha (%)	100	30	80	30
RDT (%)	100	45	70	45
MemVLA (%)	100	80	100	80

**Table 5:** Comparison of classified storage of materials task of utensils completion rates.

	Grab Garbage Bag	Open the First Drawer	Place to the First Drawer	Close the First Drawer	Identify and Grab Medicine	Open the Second Drawer	Place to the Second Drawer	Close the Second Drawer	Total
OpeVLA (%)	70	20	0	50	50	10	0	50	0
Aloha (%)	80	60	15	70	87	50	12	70	12
RDT (%)	80	55	77	100	88	67	50	90	55
MemVLA (%)	100	100	85	100	100	100	92	100	85

**Table 6:** Comparison of sorting fruit task of utensils completion rates.

	Grab the Fruit Correctly	Pick Up the Fruit Correctly	Place the Fruit Correctly	Total
OpeVLA (%)	50	100	80	50
Aloha (%)	/	/	/	/
RDT (%)	90	100	90	90
MemVLA (%)	100	100	100	100

**Table 7:** Comparison of arrangement and placement task of utensils completion rates.

	Open the First Drawer	Identify the Grasping Chopsticks	Place on the First Shelf	Identify the Grasping Spoon	Put It in a Drawer	Close the Drawer	Identify the Grasping Bowl	Place on the Second Shelf	Total
OpeVLA (%)	25	60	0	5	0	20	40	0	0
Aloha (%)	63	80	50	20	15	60	90	70	15
RDT (%)	60	80	70	40	75	90	90	80	60
MemVLA (%)	100	100	88	70	90	100	100	100	100

**Table 8:** Memory usage comparison.

	Self-Attention	Memory-Gated Filtering-Attention
Memory Usage (mb)	13,608	3754
Training time (s)	1.35	0.129

## Data Availability

All data included in this study is available upon request by contact with the corresponding author. Some collected data is not publicly available because of ethical restrictions.

## Supplementary Materials

The following supporting information can be downloaded at: https://www.mdpi.com/article/10.3390/s26010165/s1, Video S1: Put up the red square.
